# Clinical Lectures on Diseases of the Ear

**Published:** 1872-05

**Authors:** E. L. Holmes

**Affiliations:** Professor of Ophthalmology and Otology, Rush Medical College, Surgeon to Illinois Charitable Eye and Ear Infirmary


ARTICLE II. — *Clinical Lectures on Diseases of the Ear.* By E. L. HOLMES, M.D., Professor of Ophthalmology and Otology, Rush Medical College, Surgeon to Illinois Charitable Eye and Ear Infirmary.

### DIFFUSED INFLAMMATION OF THE EXTERNAL MEATUS.

*Gentlemen*—You have recently examined carefully several cases, like the one now before you, of this very important disease. In speaking of these cases, I have previously drawn your attention to their principal features.

By diffused inflammation is meant, as you are aware, an inflammation of the lining membrane of the external meatus, not confined to a small space as in furuncle, but extending usually over the greater portion of this membrane.

You have observed, on hearing the history of the cases, that the symptoms, both subjective and objective, vary much in different individuals. Pain is sometimes absolutely wanting, and sometimes excruciating. There is often simply an unpleasant irritation. There is also great difference as regards swelling. In one case you observed the soft tissues so swollen that the meatus could scarcely be examined—the tumefaction extending even to the external ear and mastoid. In other cases there was scarcely any swelling.

There is seldom absence of purulent discharge after the first few days. The poorer classes of patients, who apply for aid at the dispensary, almost invariably treat "ear-ache" with "domestic" remedies; cases without pain are wholly neglected; hence but two or three patients have applied for treatment before the discharge has appeared.

The hearing is almost invariably impaired—subjective sounds—tinnitus—appearing as the hearing begins to suffer.

In the earlier stages there is a congestion and redness of the walls of the meatus, especially near the membrana tympani, which generally extends to the membrana itself. If you have an opportunity of watching a case in this condition from day to day, you will observe that soon there is slight puffiness of the cuticle, followed by a watery discharge. The cuticle, both of the meatus and membrana, soon begins to appear white and often wrinkled. After a time the whole cuticle is thrown off, the surface becomes ulcerated, and the discharge more purulent in appearance.

The most frequent cause of this disease is exposure to changes of temperature, as in sleeping in a draught, or riding in a car by an open window. It is very frequently the result of exanthematous diseases, especially scarlatina. The repeated recurrence of furuncle in the ear sometimes leaves the meatus extensively inflamed. Burns and injuries are not an infrequent cause of this affection. In individuals of a scrofulous or syphilitic diathesis, the above exciting causes are especially active.

From your knowledge of the anatomy of the ear, the delicacy of the structures within the membrana tympani, you can readily comprehend what may be some of the results of inflammation. The membrane may be simply thickened; it may be more or less injured from perforating ulcers. In proportion as this occurs, and the inflammation extends to the middle ear, will hearing be affected. Caries of the walls of the meatus is an occasional, and polypus not an infrequent, result.

In the great majority of patients, otherwise well, the prognosis is good. Without treatment the disease must be regarded as serious. You have observed, from the history of the cases in the clinic, that the disease has been too often neglected, until the organ has been seriously injured.

The treatment, upon the whole, is very simple. In the early stages of the inflammation, in cases where there is great pain in and about the ear, with swelling and redness, a few leeches should be applied to the tragus or its vicinity, the external meatus having first been closed with cotton. Where these symptoms are not marked, local blood-letting is unnecessary. Within the first thirty-six hours, a hot bath, followed by a mild cathartic and diuretics, will often do much good. In a later stage these remedies will be less beneficial.

Of very great importance for the relief of pain is the use of warm water in and around the ear. A few drops of the water, as warm as can be well borne, should be turned into the meatus every few minutes, and cloths dipped in hot water applied over it. If there is a painful throbbing, with fever, veratrum viride or aconite, in appropriate doses, aids in giving relief. Pain may be allayed by subcutaneous injections of morphine.

As soon as the discharge commences, frequent injections of warm water should be employed to insure its removal. The purulent matter, if allowed to remain in the meatus, soon becomes decomposed and very irritating. It has often an offensive odor, which may be removed by a solution of carbolic acid or permanganate of potash.

To arrest the discharge, a few drops of a warm solution of alum or acetate of lead, ten to twenty grains to the ounce, should be turned into the ear three or four times daily. The meatus should each time be first carefully syringed with warm water and dried with a tuft of cotton on the end of a fine probe. A convenient way to warm the solution is to put a few drops of it in a teaspoon and hold it over a burning match, care being taken that they do not become too hot.

Other astringents—tannin, sulph. zinc, etc.—are often beneficial, although the two just mentioned are perhaps preferable.

If the patient is in good health, there is little necessity in this stage of the disease for internal treatment. Otherwise, as in cases of scrofulous or syphilitic diathesis, the specific remedies should be given.

The disease is especially obstinate in children living in crowded and ill-ventilated apartments, with insufficient clothing and improper food. Treatment, which is so beneficial in ordinary cases, is almost useless with such children unless they can be placed in proper hygienic relations.

Whenever the walls of the external meatus and of the membrana tympani present a red and quite rough surface, analogous to "granulated lids," a solution of nitrate of silver, ten to twenty grains to the ounce, should be carefully applied over the diseased surface by means of a tuft of cotton on the end of a probe. Great care is required that the solution be not introduced too freely.

After the granulations have disappeared, the milder astringents are sufficient.

In our next lecture, we will bring in review the various cases we have seen of disease in which affection of the membrana tympani was a complication, and will consider the anatomy and physiology of this important structure.

